# CREATING DEMENTIA-FRIENDLY COMMUNITIES IN CENTRAL FLORIDA IN THE UNITED STATES

**DOI:** 10.1093/geroni/igz038.1667

**Published:** 2019-11-08

**Authors:** Tracy Wharton, Daniel Paulson, Courtney Wagner

**Affiliations:** 1 Central Florida University, Orlando, Florida, United States; 2 University of Central Florida, Orlando, Florida, United States

## Abstract

The Dementia Care & Cure Initiative in Florida is a statewide movement to advance dementia friendly communities. With 25% of the state over the age of 65, Florida has one of the highest rates of dementia in the nation. The taskforce based in Orlando involves a partnership of representatives from social service agencies, law enforcement, healthcare providers, and research partners, as well as consumers. The task force commissioned a series of five focus groups with 43 consumers. These focus groups produced short and long-term recommendations, identifying such issues as needed training for emergency personnel and law enforcement, improving inter-provider communication, and providing culturally competent programming for a diverse region. The taskforce has been planning with the Mayor’s office and law enforcement to initiate training and support for community engagement, and planning for implementation of these goals. Recommendations from the groups and from the taskforce to community leaders will be discussed.

